# Ecological Variation in Response to Mass-Flowering Oilseed Rape and Surrounding Landscape Composition by Members of a Cryptic Bumblebee Complex

**DOI:** 10.1371/journal.pone.0065516

**Published:** 2013-06-19

**Authors:** Dara A. Stanley, Mairi E. Knight, Jane C. Stout

**Affiliations:** 1 Botany Department, School of Natural Sciences, Trinity College Dublin, Dublin, Ireland; 2 Trinity Centre for Biodiversity Research, Trinity College Dublin, Dublin, Ireland; 3 School of Biomedical and Biological Sciences, Plymouth University, Drake Circus, Plymouth, United Kingdom; University of Marburg, Germany

## Abstract

The *Bombus sensu stricto* species complex is a widespread group of cryptic bumblebee species which are important pollinators of many crops and wild plants. These cryptic species have, until now, largely been grouped together in ecological studies, and so little is known about their individual colony densities, foraging ranges or habitat requirements, which can be influenced by land use at a landscape scale. We used mass-flowering oilseed rape fields as locations to sample bees of this complex, as well as the second most common visitor to oilseed rape *B. lapidarius*, and molecular RFLP methods to distinguish between the cryptic species. We then used microsatellite genotyping to identify sisters and estimate colony densities, and related both proportions of cryptic species and their colony densities to the composition of the landscape surrounding the fields. We found *B. lucorum* was the most common member of the complex present in oilseed rape followed by *B. terrestris*. *B. cryptarum* was also present in all but one site, with higher proportions found in the east of the study area. High numbers of bumblebee colonies were estimated to be using oilseed rape fields as a forage resource, with *B. terrestris* colony numbers higher than previous estimates from non-mass-flowering fields. We also found that the cryptic species responded differently to surrounding landscape composition: both relative proportions of *B. cryptarum* in samples and colony densities of *B. lucorum* were negatively associated with the amount of arable land in the landscape, while proportions and colony densities of other species did not respond to landscape variables at the scale measured. This suggests that the cryptic species have different ecological requirements (which may be scale-dependent) and that oilseed rape can be an important forage resource for many colonies of bumblebees. Given this, we recommend sustainable management of this crop to benefit bumblebees.

## Introduction

One of the most common bumblebees in North West Europe, responsible for both crop and wild plant pollination [1], is the *Bombus sensu stricto* group, a cryptic complex of five species: *B. cryptarum, B. lucorum, B. magnus, B. terrestris* and *B. sporadicus* [2]. Although advances in the taxonomy of this group have been made [2], most ecological studies of bumblebees and the pollination services they deliver have considered these species as a single group (e.g. [3], [4]) since the workers are morphologically indistinguishable in the field [5], [6], [7]. However, this means that ecological differences between the species may have been overlooked (but see [8], [9]) rendering the pollination services delivered by, and conservation status of the species belonging to, this cryptic complex impossible to assess [10].

Over the past few decades, declines in both range and abundance have been documented for several bumblebee species in both North America and Europe, whilst other species have shown no decline, and in some cases have spread and become more abundant [3], [4], [11], [12]. It is thought that the longer tongued bumblebee species, those with later starting colony development cycles, and those at range edges and with small climatic ranges, are at most risk of decline [3], [13], driven largely by agricultural intensification [14], [15], [16]. Members of the *Bombus sensu stricto* (henceforth *B. s. str*.) cryptic complex of species are all relatively short tongued, have earlier starting colony cycles and are assumed to be ecological generalists [3], [4], and therefore may not be at the same risk of decline as some other species. The *B. s. str.* group in Ireland contains both species which are classified according to the IUCN Red List criteria as of Least Concern (*B. lucorum* and *B. terrestris*) but also species which cannot be assigned to a threat category because they are currently Data Deficient (*B. cryptarum* and *B. magnus*) [10]. However, due to the cryptic nature of these species, it is quite possible that these classifications are inappropriate as the relative proportions of these cryptic species in both semi-natural and agricultural sites are not well known.

Individuals of the *B. s. str.* group are the most commonly observed bumblebees visiting mass-flowering oilseed rape in Ireland (followed by *B. lapidarius*; Stanley & Stout unpublished data), and as the crop benefits from insect pollination [17], are likely to be important pollinators. Although it has been grown in Europe for centuries, the distribution of oilseed rape is changing and it is becoming more common largely due to its use as a bioenergy crop [18]. Since bumblebees, including the *B. s. str*. group, have large foraging ranges in comparison to other bee species ([19], for a summary of bumblebee foraging ranges see [20], [21]), and are influenced by the composition of habitats and features within landscapes at both smaller [22] and larger spatial scales [23], they may be sensitive to changes in cultivation patterns of mass-flowering crops such as oilseed rape. Bumblebee foraging distances can vary with the proportion of forage habitats in the landscape [24], and landscape scale factors can also influence colony fitness [25]. Several field surveys have demonstrated that the abundance of bumblebees can be influenced by landscape features. For example, more bees of the *B. s. str*. group (as well as *B. lapidarius* and *B. pascuorum*) were observed when surrounding landscapes (up to 3 km from the sampling site) contained a high availability of mass-flowering oilseed rape in Germany [26], while oilseed rape fields had more bumblebees of all species when there was more pasture in the surrounding landscape (at an 800 m radius) in Canada [27]. Conversely, other studies have shown no relationship between the abundance of bumblebees and landscape features [17], [22], [28]. However, many studies so far have grouped all bumblebee species together when looking at how bees are influenced by the surrounding landscape, when species-specific responses are likely, and have primarily focussed on the abundance and richness of bumblebees [26], [27].

As colonial organisms, work on the effect of the surrounding landscape at the reproductive level (on colony densities) is important in order to predict impacts on populations, and landscape scale effects on colony densities of some distinguishable species have been investigated [24], [25], [29]. Since it is notoriously difficult to find and quantify colony densities using observational methods [30], molecular techniques have been developed allowing estimations of colony densities based on the relationships of bumblebee workers or sisters to each other [31], [32], [33]. This has allowed estimation of nest or colony density and foraging distances, which differ remarkably between different bumblebee species (for a review see [20]). As the most distinguishable of the *B. s. str*. complex, with queens and some workers having a darker buff-coloured tail, *B. terrestris* has been extensively studied (although lighter coloured individuals may have been overlooked; [5]). However, colony densities, foraging distances, landscape scale effects and even distributions of the other species in the complex are not well known. This ecological information is essential to understand how to manage, protect and conserve these important pollinator species, and may help to explain their co-existence.

The aim of this study was to investigate ecological differences between species within the *B. s. str.* complex by estimating the relative abundance and colony density of each species and then relating those data to landscape composition along an agricultural landscape gradient. For comparison another non-cryptic short tongued species, *B. lapidarius* (which is second in abundance to the cryptic complex in oilseed rape fields, but designated as Near Threatened (NT) in Ireland as a whole), was also included in the study. We used mass-flowering oilseed rape fields as a sampling unit as they are commonly visited by the *B. s. str*. group and are likely to attract bumblebee colonies from the surrounding agricultural matrix. Specifically we aimed to.

1. investigate whether all of the cryptic species of the *Bombus s. str.* group are found foraging in oilseed rape fields and determine the relative abundance of each species.
2. estimate the number of colonies (colony density) of the cryptic species, and *B. lapidarius*, using oilseed rape fields as a foraging resource; and compare the number of colonies in oilseed rape fields to previously published colony densities in agricultural habitats.
3. identify ecological differences between the cryptic species of the *B. s. str*. complex in agricultural habitats by investigating whether the number of estimated colonies, and relative proportions, of the cryptic species relate differently to the composition of the landscape surrounding the oilseed rape fields.

## Methods

### Site Selection

**Figure 1:**
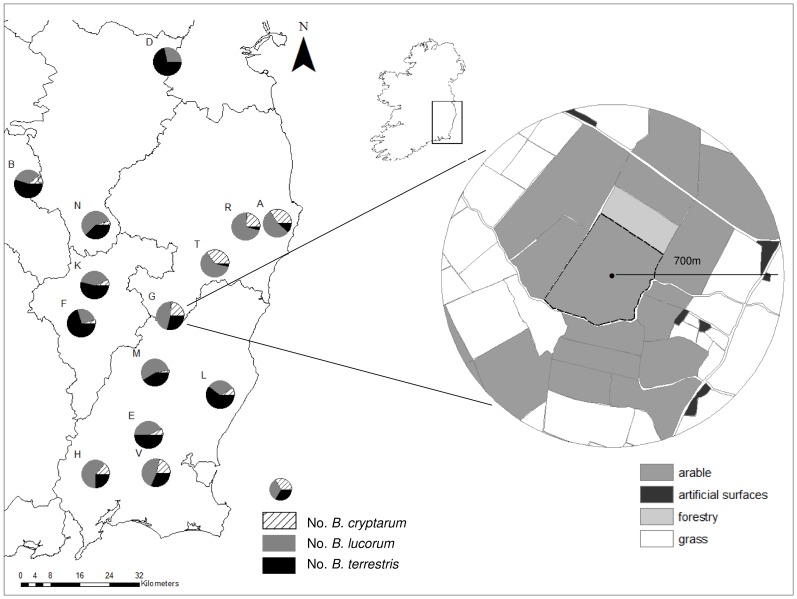
Location of the 14 spring oilseed rape fields in South East Ireland, and proportions of each of the cryptic species identified in each site. An example of the landscape mapped at a 700 m radius around each field is given, with the focal oilseed rape field highlighted with a dotted line.

Fourteen spring oilseed rape (canola, *Brassica napus* L.) fields were selected for study in an area of 114 km×62 km in South East Ireland in 2010 (Fig. 1), where beef and dairy farming are interspersed with arable, and oilseed rape is relatively rare. All fields were privately owned, and permission to sample was obtained from relevant land owners. Fields were selected along a landscape gradient of arable to pasture dominated landscapes based on CORINE land cover data [34] (Table 1). Fields were on average 15.28 km apart (range 2.9–48.2 km). Due to the current knowledge on average foraging ranges of our focal bumblebee species (for summaries of estimates of foraging distances of *B. terrestris* and *B. lapidarius* see [20], [21]; foraging distances of *B. cryptarum* and *B. lucorum* are unknown), it was assumed that given our average inter-site distance, the number of sites sharing bees from the same colony would be negligible overall.

**Table 1:** Sample sizes (A) and Capwire point estimates of number of colonies (B) of the different bee species in the different sites.

Site	Location	S (ha)	P (%)	D (km)	*B. cryptarum*		*B. terrestris*		*B. lucorum*		*B. lapidarius*	
					A	B	A	B	A	B	A	B
**A**	**Rathdrum, Co. Wicklow**	5.1	23	2.9	29	48	27	49	59	551	0	0
**B**	**Stradbally, Co. Laois**	31.5	43	21.3	10	42	73	304	32	485	0	0
D	Celbridge, Co. Kildare	6.9	34	48.2	0	0	43	211	18	n/a	44	71
**E**	**Adamstown, Co. Wexford**	3.6	11	10.5	10	19	55	95	54	698	0	0
**F**	**Bagnelstown, Co. Carlow**	7	49	11	8	n/a	63	258	31	83	51	42
G	Carnew, Co. Wicklow	12.7	58	15.9	10	n/a	17	28	27	n/a	43	38
**H**	**New Ross, Co. Wexford**	2.5	55	16.3	5	8	32	72	44	n/a	0	0
**K**	**Carlow, Co. Carlow**	5.6	63	11	9	n/a	53	135	36	303	52	48
**L**	**Kilmuckridge, Co. Wexford**	5.8	48	18.6	15	n/a	60	157	45	183	0	0
**M**	**Ballycarney, Co. Wexford**	4.6	78	15.9	5	n/a	47	119	62	357	45	84
N	Castledermot, Co, Kildare	8.7	71	16.3	3	n/a	21	203	36	198	44	40
R	Rathdrum, Co. Wicklow	4.3	11	2.9	12	n/a	2	n/a	33	253	0	0
**T**	**Aughrim, Co. Wicklow**	3.9	8	13	38	690	3	n/a	79	487	40	38
**V**	**Taghmon, Co. Wexford**	8.6	40	10.5	21	63	35	73	60	570	0	0
	**mean**	**7.9**	**42**	**15**	**13**	**145**	**38**	**142**	**44**	**379**	**46** *	**52**

* mean of sites with species present only.

(Site names in bold are those with a total of Oilseed rape and adjacent field together). S = area of focal oilseed rape field, P = % arable land in surrounding landscape, and D = distance to nearest sampled oilseed rape field, measured from the centroid of the oilseed rape field.

### Sample Collection

Fields were visited once between 13^th^ June and 12^th^ July 2010 during the peak flowering of spring oilseed rape. Firstly, two 100 m transects were walked at a slow steady pace in each site to identify the main bumblebees present. Then, individuals of the *B. s.str*. group were sampled qualitatively in each field by walking around the entire perimeter of the field on the outermost tramline (approximately 20 m into the crop) and catching individuals until a target of 60 were caught. A non-lethal sample of tarsal segment was taken from the mid-leg and stored in 75% ethanol for later DNA analysis [35]. In the seven sites where *B. lapidarius* occurred in the transects, a target of 50 *B. lapidarius* individuals were also sampled in the same way. Sampled individuals were marked using a non-toxic marker pen to avoid re-capture of the same individuals. A similar amount of time was also spent catching bees around the perimeter (field margin) of a non mass-flowering field directly adjacent to the oilseed rape field. This was initially done to investigate patterns of worker distribution from individual colonies, but very few bees from the same colonies (sisters) were found in either field, which meant that we could not investigate this further. It was only possible to sample *B. s. str.* workers in nine adjacent fields as there were no suitable sampling areas (flowering vegetation) at the other five fields. Most *B. lapidarius* individuals were primarily attracted to oilseed rape fields; individuals were rarely present in adjacent fields and so numbers were too low to include in estimates of colony density. A total of 1362 individuals from the *B. s. str.* group and 330 *B. lapidarius* individuals were sampled overall.

### Landscape Characterisation

A detailed map of the landscape surrounding each oilseed rape field up to a 700 m radius from the centroid of each field was also produced (Fig. 1). This radius was chosen based on the estimated foraging distance of the focal bee species (as reviewed in [20]). Land cover was divided into the following categories (Tables S1 & S2 in supporting information): mass-flowering crops (average across all sites: 79% oilseed rape, 14% potatoes, 7% field beans), non-mass-flowering arable land, grassland, forestry and extensively modified human surfaces (including buildings, yards and gardens). Land cover types were ground-truthed for each landscape as accurate distinction using aerial photographs was not possible. The length of field boundaries and area of fields sampled were also quantified using ortho-photographs and Ordinance Survey maps. All landscape analyses were carried out in ESRI ArcGIS 9.3.

### Species Differentiation and Microsatellite Genotyping

DNA was extracted from tarsal segments by pulverising each sample after cooling in liquid nitrogen, and adding 300 µL 10% solution of Chelex 100 heated to 80°C to each sample. Samples were then heated to 100°C for 15 minutes before centrifuging and finally cooling to 4°C. We used a Restriction Fragment Length Polymorphism (RFLP) method developed by Murray et al. [8] to definitively assign each sample to one of the cryptic species. Samples were amplified using a polymerase chain reaction (PCR), digested using specific restriction enzymes (EcoNI and HinFI) and then visualised using electrophoresis in 2% agarose gels, where each cryptic species has a unique banding pattern [8]. Sixteen individuals (including a mixture of ambiguous and confirmed banding patterns) were also sequenced at partial mitochondrial COI gene to confirm RFLP identities [36].

Samples from all four species were subsequently genotyped at 14 microsatellite loci in two multiplex reactions (all: B10, B11, B96, B100, B118, B124, B126, B132, BT08, BT11, BL02, BL06, BTERN01, *B. terrestris* and *B. lucorum*: BL03, *B. lapidarius* and *B*. *cryptarum*: BL11, Table 2) [37], [38], [39]. PCR products were visualised on an ABI 3730×l automated sequencer (Applied Biosystems) using GeneScan™ 500 LIZ® size standard, and alleles were sized using GENEMAPPER® software (Applied Biosystems). Where a sample failed to amplify at any locus on the first attempt, or where there was any case of scoring ambiguity, a new PCR was run and all loci were re-amplified. This also allowed calculation of scoring and allelic drop-out error rates for loci that were amplified twice. After two attempts, B100 still failed to amplify sufficiently for accurate scoring in both *B. terrestris* and *B. lucorum*, and B10, B96 and BT08 in *B. lapidarius,* and so these loci were omitted from any further analyses.

**Table 2:** Characteristics of the microsatellite loci used in each species.

	*B. cryptarum* *			*B. terrestris*			*B. lucorum*			*B lapidarius*		
locus	AR	Fis ± SE	N	AR	Fis ± SE	N	AR	Fis ± SE	N	AR	Fis ± SE	N
B10	1.92	0.029±0.032	22	5.35	0.073±0.02	17	8.76	−0.025±0.017	18	na	na	na
B100	1.78	0.088±0.072	10	na	na	na	na	na	na	4.53	0.008±0.061	5
B11	1.45	0.137±0.046	8	3.13	0.127±0.045	9	4.26	0.022±0.032	7	3.82	−0.057±0.049	5
B118	1.72	0.138±0.050	7	3.48	0.104±0.039	8	3.10	0.066±0.037	6	3.46	−0.015±0.054	5
B124	1.67	0.008±0.043	8	4.94	0.094±0.02	16	4.88	0.017±0.025	8	4.93	−0.041±0.032	6
B126	1.89	0.099±0.037	19	4.30	0.099±0.033	18	8.92	0.017±0.017	18	5.01	0.073±0.085	8
B132	1.8	0.013±0.027	15	4.34	0.079±0.031	13	11.33	0.019±0.014	25	4.04	0.033±0.023	5
B96	1.51	0.247±0.096	5	3.00	0.088±0.054	8	3.10	0.018±0.034	7	–	–	–
BL02	1.92	0.026±0.025	22	4.59	0.023±0.024	12	10.85	0.01±0.009	23	4.37	0.007±0.033	6
BL03	–	–	–	4.51	0.049±0.031	17	8.84	−0.024±0.017	20	–	–	–
BL06	1.93	−0.000±0.031	25	2.65	0.046±0.032	15	10.89	0.071±0.011	23	5.13	−0.022±0.029	9
BL11	1.93	0.040±0.045	24	–	–	–	–	–	–	6.03	−0.016±0.037	8
BT08	1.79	0.168±0.046	14	6.05	0.084±0.03	19	4.99	−0.005±0.02	10	–	–	–
BT11	1.85	−0.010±0.095	13	3.41	0.137±0.024	13	6.14	0.069±0.023	10	–	0.566±0.116	4
BTERN01	1.82	−0.007±0.051	13	4.67	0.063±0.019	13	5.08	−0.014±0.025	11	2.99	−0.003±0.047	4

* population N was removed from analysis of *B. cryptarum* as there were no allele scores in that population for BT11.

AR = average allelic richness across populations, Fis = inbreeding coefficient jack-knifed across populations (calculated in FSTAT) and N = total number of alleles. Minimum sample sizes for AR calculations are as follows: *B. cryptarum* = 1, *B. terrestris* = 6, *B. lucorum* = 12, *B. lapidarius* = 20. Loci identified by Estoup et al. [37], Estoup et al. [38] & Funk et al. [39].

### Genetic Data Analyses

For analysis, we included any samples with a minimum of seven of the 13 loci scored for *B. terrestris* and *B. lucorum,* and a minimum of six loci for *B. cryptarum* and *B. lapidarius*. All data were analysed on a per site basis. Genotypes were checked for typographic error and null alleles using MICRO-CHECKER [40]. We then used the program COLONY [41] to identify the number of colonies sampled in each site (colony density), using allelic drop out and scoring error rates calculated from re-scoring. COLONY implements a maximum likelihood sibship reconstruction method [42] and has been shown to give the most accurate sibship reconstruction when compared with other methods [43]. Due to the assumptions of COLONY, GENEPOP 4.1 [44] was used to test for deviations from Hardy Weinberg equilibrium (HWE) of individual loci by site using a probability test, and linkage disequilibrium between loci across all sites, using Bonferroni corrections for multiple comparisons of loci per individual. Summary characteristics of microsatellite loci were calculated using FSTAT [45] (Table 2). For GENEPOP and FSTAT analyses only one individual per colony was retained, as inclusion of closely related family members will inevitably lead to inflated homozygosity estimates that can lead to spurious deviations from HWE expectations [32], [46].

However, sampling was not exhaustive, and the COLONY estimates are based only on workers successfully sampled from each site. Therefore, to account for the number of colonies we did not sample at each site, we also estimated the total number of colonies (total colony density) present in each site for each species. To do this we used the CAPWIRE programme [47] which is a mark-recapture software that allows for multiple sampling of an individual and can also be used for estimating number of bumblebee colonies [25]. CAPWIRE implements two different estimation methods; the Even Capture Model (ECM) assumes equal chances of sampling bees from the same colony [47] and provides very similar estimates to those obtained in previous studies using the truncated Poisson method [25], while the Two Innate Rate Model (TIRM) assumes unequal rates of capture of different colonies. Although the TIRM method has been shown to be most useful for estimating the number of bumblebee colonies in other work [25], a likelihood ratio test (LRT) on our data found the ECM method to be preferable in the majority of cases (Table S3) and so we use this estimate in landscape analyses here. However, results of TIRM estimations can be found in the supporting information (Table S3). CAPWIRE models were run in 0.1 increments with capturability ratios of minimum 1, maximum 20; 95% confidence intervals for the estimate on population size based on 1000 bootstrap replicates; a largest population size of 750 for dimensioning; and a likelihood ration rejection region of 0.2 when conducting likelihood ratio tests. We estimated total colony density in each field two ways: firstly we used sisters identified in the oilseed rape field only to get an estimate of colonies using that resource. Secondly, since very low numbers of sister pairs were identified within the oilseed rape fields for both *B. cryptarum* and *B. lucorum* (and sister pairs or recaptures are necessary for further estimation using CAPWIRE or truncated Poisson methods), we pooled these data with those from the adjacent field (these pooled data henceforth referred to as “site”) for each species to increase sample sizes and number of sister pairs. This allowed us to get a more accurate estimate of the numbers of colonies foraging in the area.

We also calculated the total number of colonies per km^2^ for the two species (*B. terrestris* and *B. lapidarius*) for which estimated foraging distances have been published (*B. terrestris* 758 m, *B. lapidarius* 450 m, [33]). These calculations per km^2^ from our study (based on *B. terrestris* from 12 fields and *B. lapidarius* from 7 fields) were then compared to those from other studies (*B. terrestris* previous estimates data from a number of sources (5 data points), summarised in [20], *B. lapidarius* previous estimates data (12 data points) from [25], [23]) using non-parametric Kruskal-Wallis tests.

### Landscape Analyses

Both proportions and colony density estimates for each species were initially investigated for correlative relationships with geographic location (ITMx and y co-ordinates), while colony density estimates were also investigated for relationships with the area sampled and the number of individuals sampled using Spearmans rank correlation.

To test whether the cryptic species responded differently to the composition of the landscape surrounding the sites, generalized linear modelling was used. Proportions and total estimated colony densities (from CAPWIRE using ECM) of each species were modelled as separate response variables, and landscape composition variables were predictors. For the proportions of each cryptic species, binomial GLMs were used to account for proportional data, and corrected for overdispersion using quasi-binomial GLMs if necessary. For models of the colony density estimates of each species, Poisson GLMs were used and standard errors corrected for overdispersion using quasi-poisson errors. Landscape variables were first normalised ((variable-mean)/standard deviation), and after removing variables that were highly co-linear (see Tables S1 & S2), area of arable land, artificial surfaces, mass-flowering crops, forestry and length of field boundaries were used in the models. Significance of terms was assessed using Z tests in the multcomp package [48] which corrects for multiple comparisons, and full models are presented (simplified models are also available in Table S4). All models were validated by plotting deviance and Pearson residuals against fitted values and explanatory variables, and by normal QQ-plots [49]. For colony densities, only sites where an estimate was obtained were used in analyses (i.e. sites where no sisters were found were not used as no accurate estimate could be calculated, thus perhaps excluding those with the highest colony densities). *B. cryptarum* colony density estimates had one outlier (site T) that had a much higher estimate than all other sites (with only one sister pair found in the largest sample of this species), and so landscape analyses were carried out both including and excluding this site. All analyses were carried out using the stats package in R version 2.15.2 [50].

## Results

### Proportions of Cryptic Species

**Figure 2:**
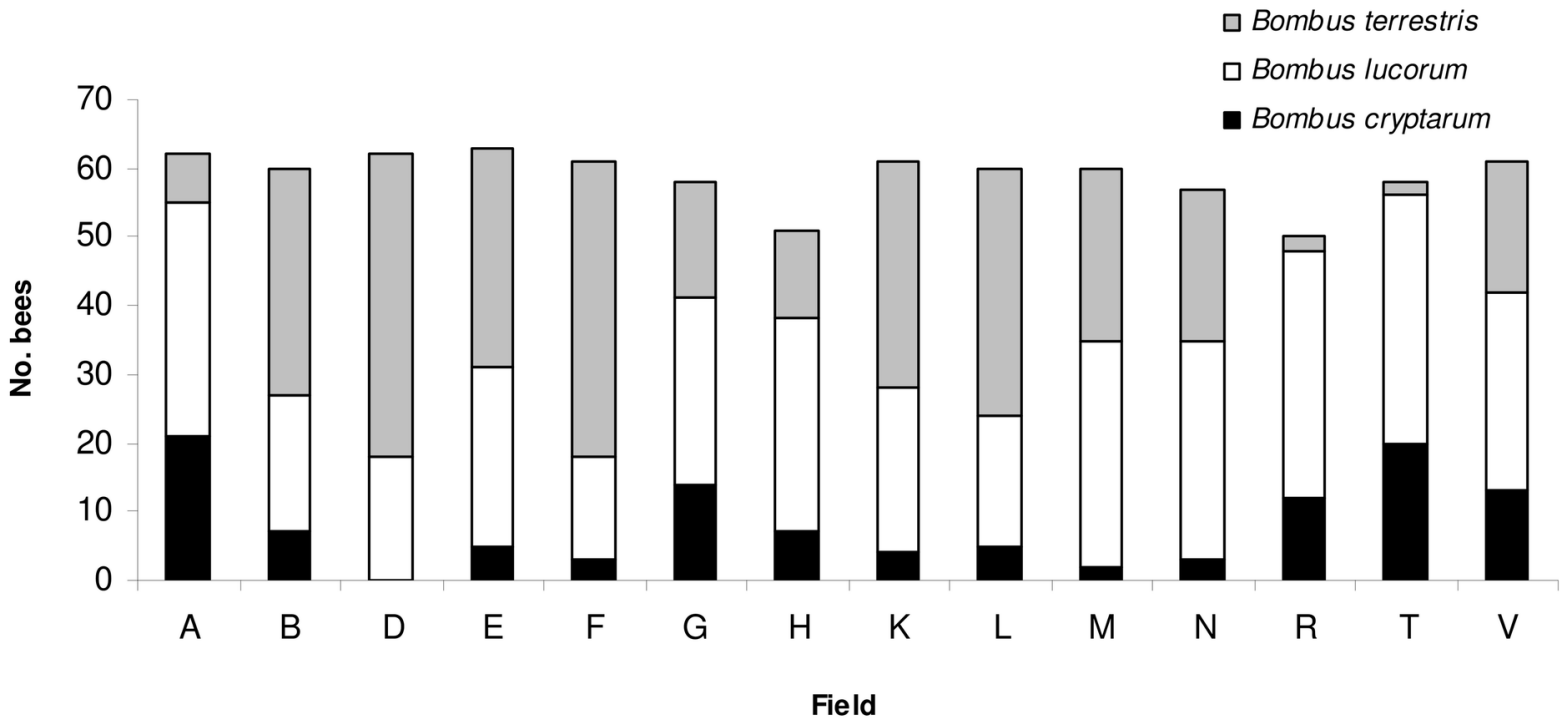
The numbers of the cryptic species of the *Bombus sensu stricto* group recorded in each oilseed rape field.

Three of the four species of the *B. s. str.* group found in Ireland were found foraging in oilseed rape fields – *B. cryptarum*, *B. lucorum* and *B. terrestris.* No *B. magnus* individuals were found in any of the fields studied. The most abundant species was *B. lucorum* (mean proportion of individuals per field 0.47±0.04 standard error), followed by *B. terrestris* (0.39±0.06). *B. cryptarum* was also present in all but one of the fields, but in lower numbers that the other two species (0.14±0.03, Fig. 2). Proportions of all three species varied among fields (Fig. 2).

### Microsatellite Genotyping

Thirteen loci were scored for *B. lucorum* and *B. terrestris*, 14 for *B. cryptarum* and 11 for *B. lapidarius*, with variation between species in terms of loci characteristics (Table 2). There were no typographical errors found using Microchecker.

For *B. lucorum* and *B. cryptarum* a global test showed no overall deviations of any loci from HWE (global Fishers test: *B lucorum* χ^2^ = 373.24, df = 364, p = 0.36, *B. cryptarum* χ^2^ = 271.16, df = 316, p = 0.97). There was no significant linkage disequilibrium detected between loci for *B. cryptarum*. Although a number of loci showed significant linkage disequilibrium using a global test across all populations (sites) for *B. lucorum,* on further investigation of each case this was driven by linkage found on one population only. Due to the small number of populations affected (1 out of 14) all loci were retained in further analyses.

For *B. lapidarius*, BT11 showed significant deviation from HWE in 3 of the 6 populations, possibly due to a deficit of heterozygotes suggesting the presence of a null allele. Therefore this locus was removed from analysis and COLONY sibships re-run without it. There were no deviations from HWE for any of the remaining loci (global Fishers test: χ^2^ = 101.18, df = 140, p = 0.99), and no significant linkage disequilibrium between any loci.

For *B. terrestris*, a global Fishers test showed significant deviation from HWE (χ^2^ = infinity, df = 310, p<0.001). However, on further investigation this was caused by a small number of loci in four populations only: BT08 in site A, B132 in site B, B126 in site F and BT11 and BT08 in site M. The following loci showed significant linkage disequilibrium but again in one population only: B132 and B124 in site B, BL03 and BL06 in site D and B126 and B118 in site E. Due to the small number of populations affected (1 or 2 out of 14 in all cases) all loci were retained in further analyses.

### Colony Estimation

Low numbers of sister pairs of all species were found within each field, suggesting that high numbers of colonies were using this mass-flowering resource (Table 1, Table S3). Most colonies were represented by a single worker, and the maximum numbers of sisters from any one colony was four (from an average of 31 individuals per site). To examine the total estimated density of colonies using mass-flowering oilseed rape fields as a resource, we first estimated colony densities using bees caught only in the oilseed rape. Colony estimations were not possible in fields where all sampled colonies are represented by one individual only, as this represents a potentially endless population; therefore estimations of total colony densities were possible in 12 fields for *B. terrestris* (mean 131, range 19–303 colonies), but only in one field for *B. cryptarum* (41 colonies), and four fields for *B. lucorum* (mean 214, range 100–303 colonies), despite similar sample sizes to previous studies (e.g. [20], [25]). *B. lapidarius* was only found in sufficient numbers in oilseed rape fields and colony estimates ranged from 38–84 colonies per field (Table 1).

Given these limitations, we also pooled data from both the oilseed rape field and adjacent field (hencefoth “site”) to estimate total colony densities, as sample sizes (and number of sister pairs) were larger, allowing estimates of total colony density using a larger number of sites (Table S3). Using an average across all sites where estimations were possible, the highest number of colonies found were of *B. lucorum,* then *B. terrestris* and then *B. cryptarum* (Fig. 3.1, Table 1). Colony densities of all species were not significantly related to the size of the fields sampled. Colony densities of *B. lucorum* and *B. lapidarius* (using only sites where colonies were sampled) were not significantly related to the number of individuals sampled, but colony densities of *B. cryptarum* (Spearmans rank correlation: Rho = 0.86, S = 8, p = 0.02) and *B. terrestris* (Spearmans rank correlation: Rho = 0.85, S = 44, p<0.001) were, suggesting that larger sample sizes may have detected more colonies. In addition, differences in allelic richness between species may indicate differences in power of resolution between species (Table 2). Given these factors, actual values of estimates (rather than patterns) should not be over-interpreted.

**Figure 3:**
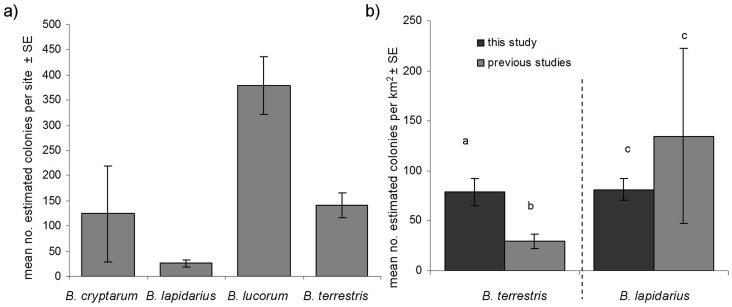
Mean numbers of colonies estimated foraging per field (a) and mean colony density per km^2^ in comparison with previous work (b). Mean colony density per km^2^ of *Bombus terrestris* was calculated using a foraging range of 758 m (n (our study) = 12, n (previous studies) = 5), and *B. lapidarius* using a foraging range of 450 m (n (our study) = 7, n (previous studies) = 12), and are provided in comparison to previous estimates (*B. lapidarius* previous estimates data from Goulson et al. [25] and Knight et al. [31]. *B. terrestris* previous estimates data from a number of sources, summarised in Charman et al., [20]). Letters indicate significant differences (p<0.05) determined using Kruskal-Wallis tests.

Using total colony densities calculated per km^2^ (Table S3), we found density of colonies of *B. terrestris* from our samples using oilseed rape fields to be significantly higher than previously published estimates (Kruskal-Wallis χ^2^ = 4.9, df = 1, p = 0.027, n (our study) = 12, n (previous studies) = 5, Fig. 3), while colonies of *B. lapidarius* were not (Kruskal-Wallis χ^2^ = 3.46, df = 1, p = 0.063, n (our study) = 7, n (previous studies) = 12, Fig. 3). Comparisons for *B. cryptarum* and *B. lucorum* were not possible as, to our knowledge, there are no previously published estimates of colony densities for these species.

### Landscape Analyses

**Figure 4:**
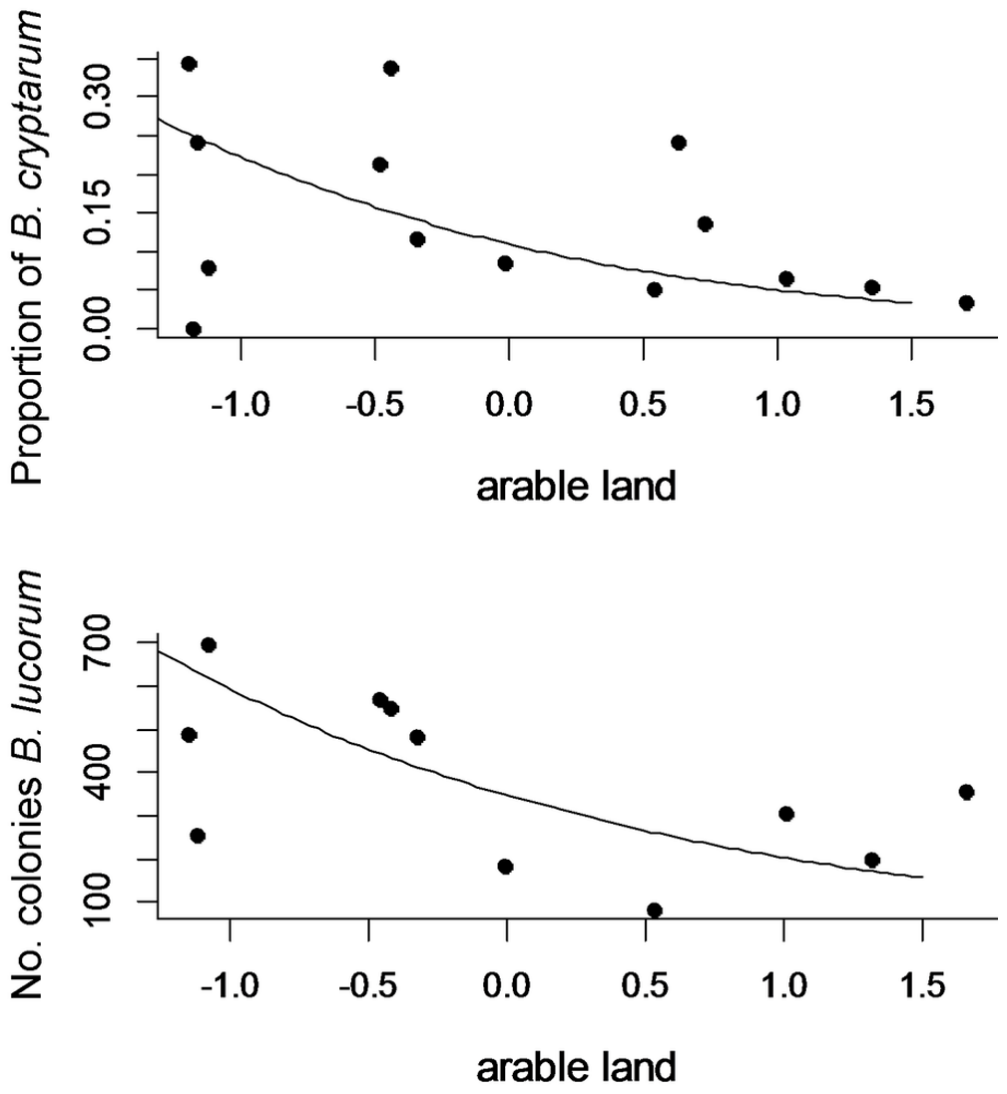
Negative relationships between amount of arable land in a 700 m radius and a) proportions of *B. cryptarum* and b) colony densities of *B. lucorum*. Points show normalised measured values, and lines show model predictions.

Proportions of *B. cryptarum* were positively related to longitude (Spearmans rank correlation: S = 206, p = 0.046, Rho = 0.55); higher proportions of the species were found in the eastern part of the study area (Fig. 1), where there were also fewer *B. terrestris* found (Spearmans rank correlation: S = 686, p = 0.07, Rho = −0.51). Proportions of *B. cryptarum* were negatively associated with the amount of arable land (Table 3., Fig. 4), while proportions of the other cryptic species were not related to landscape compositional variables at the 700 m radius studied (Table S5). Colony densities of *B. lucorum* were also negatively related to the amount of arable land in the landscape (Table 3, Fig. 4), while other colony densities were not related to landscape variables (Table S5).

**Table 3:** Results of multiple comparisons of variables from generalized linear models investigating the effects of landscape composition measured within a 700 m radius on proportions and colony densities of each species.

	estimate	z-value	Pr(>|z|)	df	Model fit
**Proportion ** ***B. cryptarum***				8	0.66
Arable land (area)	−0.85	−2.94	0.02*		
Forestry (area)	0.18	0.58	0.97		
Mass-flowering crops (area)	−0.62	−1.91	0.22		
Artificial surfaces (area)	−0.85	−1.74	0.31		
Field boundaries (length)	−0.20	−0.69	0.95		
**Colony density ** ***B. lucorum***				5	0.65
Arable land (area)	−0.54	−2.64	0.04*		
Forestry (area)	−0.09	−0.31	0.99		
Mass-flowering crops (area)	−0.12	−0.69	0.93		
Artificial surfaces (area)	−0.38	−1.33	0.54		
Field boundaries (length)	0.11	0.47	0.98		

Only models with significant components (marked with a *) are displayed. Proportion estimates are on the logit scale, and colony estimates on log scale. Model fit is calculated as follows: ((null deviance – residual deviance)/null deviance) [49].

## Discussion

Bumblebee communities are often composed of a number of morphologically similar species, including members of the cryptic *B. s. str* complex. However, subtle ecological differences may exist among species, which have been overlooked in the majority of previous studies which have grouped them together. We have shown that three members of the *B. s str.* complex co-exist in mass-flowering fields in Irish farmland, with *B. lucorum* most ubiquitously abundant (in accordance with other studies in different habitats; [8], [51]), but with *B. terrestris* and *B. cryptarum* also common. The fourth species of the complex, *B. magnus*, was not observed, but previous work has suggested that *B. magnus* is an upland species associated with heathlands and Ericaceous species [3], [9]. The four species observed differed in colony densities and responses to surrounding landscape, suggesting the species are ecologically distinct and possibly explaining their ability to co-exist. In addition, we found that the numbers of colonies of bumblebees using resources in oilseed rape fields was particularly high. Our data indicate that *B. cryptarum* is widespread in Ireland and is certainly under-recorded due to its morphological similarity to the other cryptic species. Meanwhile, the distribution of *B. lapidarius* in agricultural areas in Ireland appears to be patchy; it was locally abundant in some fields, and absent from others.

As resources for pollinators become increasingly sparse in agricultural areas, mass-flowering crops can have positive impacts on bumblebee abundance [26], [52] and on colony growth [53], [54], [55]. Here we also show that not only large numbers of individual bees use mass-flowering fields, but that they come from a large number of different colonies; we estimated between 648–831 colonies of the species studied using a site containing oilseed rape. Assuming all nests are located within the 700 m landscape radius measured, a colony would be located on average every 20–45 m of field boundary (although some colonies may be coming from further afield).

Bumblebees have been shown to vary their foraging distance based on the availability of resources in their environment [24], [56]. We estimated significantly more colonies of *B. terrestris* per km^2^ in our study than in previous work, suggesting that either a) there is a higher background number of *B. terrestris* colonies in Ireland than in the UK and Germany where previous studies have been carried out (perhaps as the intensity of land use in Ireland is less, which can support higher densities of *B. terrestris* [57]), or b) that *B. terrestris* will fly longer distances to exploit a mass-flowering crop, therefore inflating the colony density estimates using the site. *B. terrestris* has been found to be able to fly long distances on occasion [58] and to quickly complete its colony cycle when growing next to a mass-flowering crop [55], suggesting that mass-flowering crops can be exploited by this species and at long distances. Interestingly, we found no difference between our estimates of colony density of *B. lapidarius* and previous estimates from other studies. *B. lapidarius* was only found foraging in oilseed rape at most sites, and not in the surrounding landscape. This suggests that *B. lapidarius* individuals within the vicinity of a mass-flowering resource will use that resource relatively exclusively.

Although mass-flowering oilseed rape fields can provide forage resources for a large number of bumblebee colonies in agricultural areas, not all bumblebee species will respond in the same way. As species studied here are the most abundant visitors to oilseed rape and are short tongued, oilseed rape may provide a disproportional benefit to these species, which are already common in agricultural areas. This in turn could have consequences for more specialised long-tongued species and for their interactions with flowering plants [59].

Although our sample sizes were similar to those used in previous studies (e.g. [20], [25], [29]), the majority of colonies were represented by single individuals. In some sites, no sister pairs were identified, preventing any total colony density estimations. Therefore, the estimates of colony densities have large confidence intervals (Table S3) [47]. However we think it is most likely that our colony density estimations are conservative for three reasons: 1) as we had to exclude sites with no sisters from further analyses, we most likely excluded sites with larger numbers of colonies that we were not able to detect, 2) estimations of colonies were based on bees found in an oilseed rape field and in most cases an adjacent field also; therefore, some sisters pairs were found outside the oilseed rape field itself which lowers confidence intervals of estimates, but may also lower estimates of colony densities using a mass-flowering resource, and 3) colony density sestimations of *B. cryptarum* and *B. terrestris* were both related to the number of individuals sampled; therefore more sampled individuals could have increased colony density estimates.

Many different factors can explain the co-existence of species in mutualist guilds [60]. Traditionally, the co-existence of bumblebees has been explained by floral resource partitioning according to tongue length [61]. Members of the *B. s. str* cryptic complex are, however, all short tongued and here we report that three species of the cryptic complex co-exist in considerable numbers in farmland; therefore factors other than tongue length must explain their co-existence. Although differences in nesting resources or foraging strategies may explain the co-existence of the cryptic species, we also found proportions of *B. cryptarum* to respond to the amount of arable land at a 700 m scale while *B. terrestris* and *B. lucorum* did not respond to any landscape measures. This suggests differences among the species in terms of their foraging ranges, and that both *B. lucorum* and *B. terrestris* may utilise the landscape at larger spatial scales than measured in this study [23], or fly further distances to access a mass-flowering resource [24], [56]. Therefore co-existence in these cryptic bumblebees may also be driven by spatial resource usage patterns [23].

More colonies of *B. lucorum* were also found with decreasing amount of arable land although proportions of this species were not related to this character. However, the colony density estimates of *B. terrestris* and *B. cryptarum* in this study were correlated with the number of individuals sampled; this suggests that adding more individuals to the sample would increase estimates, and therefore the lack of landscape associations with estimates for these species in particular should be interpreted with caution.

### Conclusions

Although species of the *B. s. str*. cryptic complex co-exist on Irish farmland, we found differences in their relative proportions and colony densities possibly related to the differential impacts of landscape on these species and *B. lapidarius,* and in turn suggesting that they may have different ecological requirements. For example, *B. cryptarum* was less common in landscapes with more arable land. This knowledge may help conservation efforts targeted to conserve this species, or may help to predict the distributions of the cryptic species which are not well known. We also found large numbers of bumblebee colonies using oilseed rape fields as a resource. This suggests that mass-flowering crops provide important forage for pollinators within agricultural areas, but it also highlights the possible severity of any negative effects of pesticides on bumblebee populations [14], and the need for sustainable management of this crop.

## Supporting Information

Table S1 **Summary of landscapes variables calculated surrounding each of the 14 fields.**
(DOC)

Table S2 **Summary of Spearmans rank correlations between compositional landscape variables describing landscapes within a 700 m radius from the focal oilseed rape field.** Top panel = Test statistic (S) and p value. Lower panel = Rho correlation co-efficient. P-values in bold with a * are significant after Bonferroni corrections for multiple tests (p<0.006). MFC = mass flowering crops, FB length = length of field boundary.
(DOC)

Table S3 **Colony density estimations for a)** ***Bombus terrestris*** **, b)** ***B. lucorum*** **, c)** ***B. cryptarum*** **and d)** ***B. lapidarius*** **at each site.** N_ind_ = number of individual worker bees sampled, N_sis_ = total number of sister pairs found within the sampled individuals using COLONY analyses of microsatellite data, and in the oilseed rape field only (OS), adjacent field only (ADJ) and shared between the two (Shared). N_obs_ = number of colonies observed, based on sibship reconstruction from Colony. N_tot_ = total number of colonies estimated, including un-sampled ones, using TIRM or ECM methods in CAPWIRE. ECM methods are equivalent to previously used truncated Poisson methods [25]. A likelihood ratio test (LRT) was also used to compare between models for each sample, and best model is shown here; this was not used in the main text as it is sensitive to small sample sizes [47], but is given here for comparison with previous work. Values with no upper limit, or where estimates were not possible due to an absence of sister pairs (or no re-captures), are marked “n/a”. CAPWIRE models were run in 0.1 increments with capturability ratios of minimum 1, maximum 20; 95% confidence intervals for the estimate on population size based on 1000 bootstrap replicates; a largest population size of 750 for dimensioning; and a likelihood ratio rejection region of 0.2 when conducting likelihood ratio tests. N_km_ = number of colonies estimated per km^2^ based on ECM estimations and foraging distances from Knight et al. [33].
(DOC)

Table S4 **Final generalized linear models describing the effects of landscape composition variables on proportions and colony density estimates of each species, simplified from a full model which included: area of arable land, forestry, mass flowering crops, artificial surfaces and length of field boundary.** Model fit is calculated as follows: ((null deviance – residual deviance)/null deviance) [49].
(DOC)

Table S5 **Full results of multiple comparisons of variables from generalized linear models investigating the effects of landscape composition variables on proportions and colony densities of each species.** Proportion estimates are on the logit scale, and colony density estimates are on the on log scale. Model fit is calculated as follows: ((null deviance – residual deviance)/null deviance) [49].
(DOC)
