# Supplementary material for: Ecological Variation in Response to Mass-Flowering Oilseed Rape and Surrounding Landscape Composition by Members of a Cryptic Bumblebee Complex
Source: PLoS One. 2013 Jun 19;8(6):e65516. doi: 10.1371/journal.pone.0065516 (PMC3686753; doi:10.1371/journal.pone.0065516)
Supplement: Table S1 — Summary of landscapes variables calculated surrounding each of the 14 fields. (DOC) [file pone.0065516.s001.doc]

|  | **Mean percentage ± SE (min – max)** |
| --- | --- |
| Intensive grassland | 46 ± 5 (17-74) |
| Arable land | 42 ± 6 (8-78) |
| Forestry | 6 ± 2 (0-22) |
| Mass flowering crops | 12 ± 2 (3-34) |
| Artificial surfaces | 6 ± 1 (1-21) |
|  |  |
| Surrounding field size (ha) | 6 ± 1 (2-15) |
