# Supplementary material for: Ecological Variation in Response to Mass-Flowering Oilseed Rape and Surrounding Landscape Composition by Members of a Cryptic Bumblebee Complex
Source: PLoS One. 2013 Jun 19;8(6):e65516. doi: 10.1371/journal.pone.0065516 (PMC3686753; doi:10.1371/journal.pone.0065516)
Supplement: Table S2 — Summary of Spearmans rank correlations between compositional landscape variables describing landscapes within a 700 m radius from the focal oilseed rape field. Top panel = Test statistic (S) and p value. Lower panel = Rho correlation co-efficient. P-values in bold with a * are significant after Bonferroni corrections for multiple tests (p<0.006). MFC = mass flowering crops, FB length = length of field boundary. (DOC) [file pone.0065516.s002.doc]

|  | **arable land** | **forestry** | **grassland** | **MFC** | **artificial** | **field size** | **FB length** |
| --- | --- | --- | --- | --- | --- | --- | --- |
| **arable land** | ----- | 634, p=0.16 | 824, **p*=0.001** | 700, p=0.05 | 684, p=0.07 | 398, p=0.67 | 416, p=0.77 |
| **forestry** | -0.39 | ----- | 312, p=0.38 | 408, p=0.72 | 391, p=0.63 | 788, **p*=0.003** | 411, p=0.74 |
| **grassland** | -0.81 | 0.31 | ----- | 350, p=0.43 | 368, p=0.51 | 492, p=0.79 | 406, p=0.71 |
| **MFC** | -0.54 | 0.1 | 0.23 | ----- | 458, p=0.99 | 270, p=0.15 | 740, p=0.02 |
| **artificial** | -0.5 | 0.14 | 0.19 | -0.01 | ----- | 614, p=0.22 | 264, p=0.14 |
| **field size** | 0.13 | -0.73 | -0.08 | 0.41 | -0.35 | ----- | 676, p=0.08 |
| **FB length** | 0.09 | 0.1 | 0.11 | -0.63 | 0.42 | -0.49 | ----- |
