# Supplementary material for: Ecological Variation in Response to Mass-Flowering Oilseed Rape and Surrounding Landscape Composition by Members of a Cryptic Bumblebee Complex
Source: PLoS One. 2013 Jun 19;8(6):e65516. doi: 10.1371/journal.pone.0065516 (PMC3686753; doi:10.1371/journal.pone.0065516)
Supplement: Table S3 — Colony density estimations for a) Bombus terrestris , b) B. lucorum , c) B. cryptarum and d) B. lapidarius at each site. Nind = number of individual worker bees sampled, Nsis = total number of sister pairs found within the sampled individuals using COLONY analyses of microsatellite data, and in the oilseed rape field only (OS), adjacent field only (ADJ) and shared between the two (Shared). Nobs = number of colonies observed, based on sibship reconstruction from Colony. Ntot = total number of colonies estimated, including un-sampled ones, using TIRM or ECM methods in CAPWIRE. ECM methods are equivalent to previously used truncated Poisson methods [25]. A likelihood ratio test (LRT) was also used to compare between models for each sample, and best model is shown here; this was not used in the main text as it is sensitive to small sample sizes [47], but is given here for comparison with previous work. Values with no upper limit, or where estimates were not possible due to an absence of sister pairs (or no re-captures), are marked “n/a”. CAPWIRE models were run in 0.1 increments with capturability ratios of minimum 1, maximum 20; 95% confidence intervals for the estimate on population size based on 1000 bootstrap replicates; a largest population size of 750 for dimensioning; and a likelihood ratio rejection region of 0.2 when conducting likelihood ratio tests. Nkm = number of colonies estimated per km2 based on ECM estimations and foraging distances from Knight et al. [33]. (DOC) [file pone.0065516.s003.doc]

| *a) B. terrestris* |  |  |  |  |  | |  |  |  |  | |  |  | |  |
| --- | --- | --- | --- | --- | --- | --- | --- | --- | --- | --- | --- | --- | --- | --- | --- |
| **Site** | **N_ind_** | **N_sis_** | **N_sis_ OS** | **N_sis_ Adj** | **N_sis_ Shared** | **N_obs_** | | **N_tot_ TIRM**  (low CI-high CI) | **N_tot_ ECM**  (low CI-high CI) | | **N_tot_ LRT**  (low CI-high CI**)** | **N_km_** | |  | |
| A | 27 | 7 | 1 | 4 | 2 | | 21 | 61 (30-119) | 49 (30-108) | 49 (26-108) ECM | | 27 |  | |  |
| B | 73 | 8 | 3 | 0 | 5 | | 65 | 332 (182-534) | 304 (177-633) | 304 (177-633) ECM | | 168 |  | |  |
| D | 43 | 4 | 4 | 0 | 0 | | 39 | 229 (107-610) | 211 (114-437) | 211 (114-437) ECM | | 117 |  | |  |
| E | 55 | 15 | 4 | 2 | 9 | | 42 | 116 (70-172) | 95 (63-167) | 95 (63-167) ECM | | 53 |  | |  |
| F | 63 | 9 | 7 | 1 | 1 | | 56 | 329 (164-661) | 258 (141-630) | 329 (164-661) TIRM | | 143 |  | |  |
| G | 17 | 5 | 5 | 0 | 0 | | 13 | 37 (15-68) | 28 (13-62) | 37 (14-92) TIRM | | 16 |  | |  |
| H | 32 | 6 | 3 | 4 | 0 | | 26 | 81 (42-143) | 72 (38-155) | 72 (44-155) ECM | | 40 |  | |  |
| K | 53 | 9 | 5 | 3 | 1 | | 44 | 150 (86-232) | 135 (80-258) | 135 (80-258) ECM | | 75 |  | |  |
| L | 60 | 10 | 2 | 0 | 8 | | 50 | 174 (102-297) | 157 (97-334) | 157 (97-334) ECM | | 87 |  | |  |
| M | 47 | 11 | 2 | 7 | 2 | | 39 | 152 (80-275) | 119 (74-255) | 152 (79-244) TIRM | | 66 |  | |  |
| N | 21 | 1 | 1 | 0 | 0 | | 20 | 213 (58-750) | 203 (63-203) | 203 (63-203) ECM | | 112 |  | |  |
| R | 2 | 0 | 0 | 0 | 0 | | 2 | n/a | n/a | n/a | | n/a |  | |  |
| T | 3 | 0 | 0 | 0 | 0 | | 3 | n/a | n/a | n/a | | n/a |  | |  |
| V | 35 | 7 | 2 | 3 | 2 | | 28 | 81 (44-150) | 73 (42-187) | 73 (42-137) ECM | | 40 |  | |  |
| **Mean** |  |  |  |  |  | |  | **163** | **142** |  | | 79 |  | |  |

| *b) B. lucorum* |  |  |  |  |  |  |  |  |  |
| --- | --- | --- | --- | --- | --- | --- | --- | --- | --- |
| **Site** | **N_ind_** | **N_sis_** | **N_sis_ OS** | **N_sis_ Adj** | **N_sis_ Shared** | **N_obs_** | **N_tot_ TIRM**  (low CI-high CI) | **N_tot_ ECM**  (low CI-high CI) | **N_tot_ LRT**  (low CI-high CI) |
| A | 59 | 3 | 0 | 3 | 0 | 56 | 580 (228-750) | 551 (225-551) | 551 (225-551) ECM |
| B | 32 | 1 | 0 | 1 | 0 | 31 | 502 (143-750) | 485 (155-485) | 485 (155-485) ECM |
| D | 18 | 0 | 0 | 0 | 0 | 18 | n/a | n/a | n/a |
| E | 54 | 2 | 0 | 2 | 0 | 52 | 726 (241-750) | 698 (268-698) | 698 (268-698) ECM |
| F | 31 | 6 | 1 | 3 | 2 | 26 | 103 (48-237) | 83 (41-222) | 103 (50-237) TIRM |
| G | 27 | 0 | 0 | 0 | 0 | 27 | n/a | n/a | n/a |
| H | 44 | 0 | 0 | 0 | 0 | 44 | n/a | n/a | n/a |
| K | 36 | 2 | 0 | 0 | 2 | 34 | 320 (125-750) | 303 (114-618) | 303 (114-618) ECM |
| L | 45 | 5 | 0 | 2 | 3 | 40 | 200 (98-502) | 183 (95-480) | 183 (95-480) ECM |
| M | 62 | 6 | 0 | 5 | 1 | 57 | 428 (197-750) | 357 (189-610) | 357 (189-610) ECM |
| N | 36 | 3 | 3 | 0 | 0 | 33 | 213 (86-637) | 198 (93-618) | 198 (78-618) ECM |
| R | 33 | 2 | 2 | 0 | 0 | 31 | 269 (94-750) | 253 (94-517) | 253 (94-517) ECM |
| T | 79 | 6 | 2 | 2 | 2 | 73 | 522 (267-750) | 487 (253-744) | 487 (253-744) ECM |
| V | 60 | 3 | 0 | 1 | 2 | 57 | 599 (236-750) | 570 (233-570) | 570 (233-570) ECM |
|  |  |  |  |  |  |  | 406 | 379 |  |

| *c) B. cryptarum* | |  | |  | |  | |  | |  | |  | |  | |  | |  | |
| --- | --- | --- | --- | --- | --- | --- | --- | --- | --- | --- | --- | --- | --- | --- | --- | --- | --- | --- | --- |
| **Site** | | **N_ind_** | | **N_sis_** | | **N_sis_ OS** | | **N_sis_ Adj** | | **N_sis_ Shared** | | **N_obs_** | | **N_tot_ TIRM**  (low CI-high CI) | | **N_tot_ ECM**  (low CI-high CI) | | **N_tot_ LRT**  (low CI-high CI) | |
| A | 29 | | 2 | | 4 | | 0 | | 2 | | 22 | | 53 (27-101) | | 48 (30-126) | | 48 (30-92) ECM | |  |
| B | 10 | | 1 | | 0 | | 0 | | 1 | | 9 | | 46 (13-750) | | 42 (12-42) | | 42 (12-42) ECM | |  |
| D | 0 | | 0 | | 0 | | 0 | | 0 | | 0 | | 0 | | 0 | | 0 | |  |
| E | 10 | | 3 | | 0 | | 1 | | 2 | | 8 | | 30 (9-750) | | 19 (8-42) | | 30 (8-750) TIRM | |  |
| F | 8 | | 0 | | 0 | | 0 | | 0 | | 8 | | n/a | | n/a | | n/a | |  |
| G | 10 | | 0 | | 0 | | 0 | | 0 | | 10 | | n/a | | n/a | | n/a | |  |
| H | 5 | | 1 | | 0 | | 0 | | 1 | | 4 | | 10 (4-750) | | 8 (4-8) | | 8 (4-8) ECM | |  |
| K | 9 | | 0 | | 0 | | 0 | | 0 | | 9 | | n/a | | n/a | | n/a | |  |
| L | 15 | | 0 | | 0 | | 0 | | 0 | | 15 | | n/a | | n/a | | n/a | |  |
| M | 5 | | 0 | | 0 | | 0 | | 0 | | 5 | | n/a | | n/a | | n/a | |  |
| N | 3 | | 0 | | 0 | | 0 | | 0 | | 3 | | n/a | | n/a | | n/a | |  |
| R | 12 | | 0 | | 0 | | 0 | | 0 | | 12 | | n/a | | n/a | | n/a | |  |
| T | 38 | | 1 | | 0 | | 1 | | 0 | | 37 | | 710 (203-750) | | 690 (163-690) | | 690 (163-690) ECM | |  |
| V | 21 | | 3 | | 0 | | 2 | | 1 | | 18 | | 70 (29-213) | | 63 (28-203) | | 63 (28-203) ECM | |  |
| mean |  | |  | |  | |  | |  | |  | | 153 | | 145 | |  | |  |

| *d) B. lapidarius* |  |  |  |  |  |  |  |  |
| --- | --- | --- | --- | --- | --- | --- | --- | --- |
| **Site** | **N_ind_** | **N_sis_** | **N_obs_** | **N_tot_ TIRM**  (low CI-high CI) | **N_tot_ ECM**  (low CI-high CI) | **N_tot_ LRT**  (low CI-high CI) | **N_km_** |  |
| A | 0 | 0 | 0 | 0 | 0 |  | 0 |  |
| B | 0 | 0 | 0 | 0 | 0 |  | 0 |  |
| D | 44 | 11 | 33 | 78 (47-122) | 71 (44-120) | 71 (48-120) ECM | 112 |  |
| E | 0 | 0 | 0 | 0 | 0 | 0 | 0 |  |
| F | 51 | 26 | 30 | 48 (32-69) | 42 (32-57) | 42 (32-57) ECM | 66 |  |
| G | 43 | 19 | 26 | 41 (26-61) | 38 (27-49) | 38 (27-54) ECM | 60 |  |
| H | 0 | 0 | 0 | 0 | 0 | 0 | 0 |  |
| K | 52 | 25 | 32 | 58 (36-79) | 48 (34-65) | 48 (36-65) ECM | 75 |  |
| L | 0 | 0 | 0 | 0 | 0 | 0 | 0 |  |
| M | 45 | 13 | 35 | 105 (59-164) | 84 (50-150) | 84 (50-150) ECM | 132 |  |
| N | 44 | 21 | 27 | 48 (30-68) | 40 (29-57) | 40 (29-57) ECM | 63 |  |
| R | 0 | 0 | 0 | 0 | 0 | 0 | 0 |  |
| T | 40 | 20 | 25 | 48 (28-69) | 38 (26-51) | 38 (26-51) ECM | 60 |  |
| V | 0 | **0** | 0 | 0 | 0 | 0 | 0 |  |
| mean | |  |  | 61* | 52* |  | 81* |  |

* mean of sites where B. lapidarius was present only
