# Supplementary material for: Ecological Variation in Response to Mass-Flowering Oilseed Rape and Surrounding Landscape Composition by Members of a Cryptic Bumblebee Complex
Source: PLoS One. 2013 Jun 19;8(6):e65516. doi: 10.1371/journal.pone.0065516 (PMC3686753; doi:10.1371/journal.pone.0065516)
Supplement: Table S4 — Final generalized linear models describing the effects of landscape composition variables on proportions and colony density estimates of each species, simplified from a full model which included: area of arable land, forestry, mass flowering crops, artificial surfaces and length of field boundary. Model fit is calculated as follows: ((null deviance – residual deviance)/null deviance) [49]. (DOC) [file pone.0065516.s004.doc]

|  | **Estimate** | **SE** | t | **p** | **df** | **model fit** |
| --- | --- | --- | --- | --- | --- | --- |
| **proportion *B. cryptarum*** |  |  |  |  |  |  |
| intercept | -1.9779 | 0.2295 | -8.618 | <0.001 | 11 | 0.46 |
| Arable land (area) | -0.733 | 0.2744 | -2.672 | **0.0217** |  |  |
| Artificial land (area) | -0.6862 | 0.3016 | -2.275 | **0.0439** |  |  |
|  |  |  |  |  |  |  |
| **proportion *B. lucorum*** |  |  |  |  |  |  |
| intercept | -0.1557 | 0.1493 | -1.042 | 0.316 | 13 | na |
|  |  |  |  |  |  |  |
| **proportion *B. terrestris*** |  |  |  |  |  |  |
| intercept | -0.4136 | 0.2515 | -1.645 | 0.124 | 13 | na |
|  |  |  |  |  |  |  |
| **total colonies *B. cryptarum**** |  |  |  |  |  |  |
| intercept | 3.4 | 0.34 | 10.1 | <0.001 | 5 | na |
|  |  |  |  |  |  |  |
| **total colonies *B. lapidarius*** |  |  |  |  |  |  |
| intercept | 2.65 | 0.52 | 5.11 | <0.001 | 13 | 0.45 |
| Forestry (area) | -1.46 | 0.69 | -2.12 | **0.055** |  |  |
|  |  |  |  |  |  |  |
| **total colonies *B. lucorum*** |  |  |  |  |  |  |
| intercept | 5.94 | 0.15 | 38.7 | <0.001 | 10 | na |
|  |  |  |  |  |  |  |
| **total colonies *B. terrestris*** |  |  |  |  |  |  |
| intercept | 4.96 | 0.18 | 28.15 | <0.001 | 11 | na |

*These results are from a model minus the outlier
