# Supplementary material for: Ecological Variation in Response to Mass-Flowering Oilseed Rape and Surrounding Landscape Composition by Members of a Cryptic Bumblebee Complex
Source: PLoS One. 2013 Jun 19;8(6):e65516. doi: 10.1371/journal.pone.0065516 (PMC3686753; doi:10.1371/journal.pone.0065516)
Supplement: Table S5 — Full results of multiple comparisons of variables from generalized linear models investigating the effects of landscape composition variables on proportions and colony densities of each species. Proportion estimates are on the logit scale, and colony density estimates are on the on log scale. Model fit is calculated as follows: ((null deviance – residual deviance)/null deviance) [49]. (DOC) [file pone.0065516.s005.doc]

|  | estimate | z-value | Pr(>|z|) | df | Model fit |
| --- | --- | --- | --- | --- | --- |
| **proportion *B. terrestris*** |  |  |  | 8 | 0.55 |
| Arable land (area) | 0.65 | 1.73 | 0.30 |  |  |
| Forestry (area) | -0.08 | -0.25 | 0.99 |  |  |
| Mass-flowering crops (area) | 1.10 | 2.22 | 0.11 |  |  |
| Artificial surfaces (area) | -0.01 | -0.02 | 0.99 |  |  |
| Field boundaries (length) | 0.60 | 1.55 | 0.40 |  |  |
|  |  |  |  |  |  |
| **proportion *B. lucorum*** |  |  |  | 8 | 0.52 |
| Arable land (area) | -0.13 | -0.65 | 0.95 |  |  |
| Forestry (area) | 0.10 | 0.55 | 0.97 |  |  |
| Mass-flowering crops (area) | -0.59 | -2.35 | 0.08 |  |  |
| Artificial surfaces (area) | 0.12 | 0.62 | 0.96 |  |  |
| Field boundaries (length) | -0.33 | -1.65 | 0.35 |  |  |
|  |  |  |  |  |  |
| **Colony density *B. terrestris*** |  |  |  | 6 | 0.25 |
| Arable land (area) | 0.22 | 0.61 | 0.96 |  |  |
| Forestry (area) | -0.09 | -0.38 | 0.99 |  |  |
| Mass-flowering crops (area) | 0.53 | 1.01 | 0.77 |  |  |
| Artificial surfaces (area) | -0.06 | -0.21 | 0.99 |  |  |
| Field boundaries (length) | 0.26 | 0.70 | 0.93 |  |  |
|  |  |  |  |  |  |
| **Colony density *B. cryptarum**** |  |  |  | 2 | 0.57 |
| Arable land (area) | -1.25 | -1.04 | 0.64 |  |  |
| Mass flowering crops (area) | 0.90 | 0.28 | 0.99 |  |  |
| Artificial surfaces (area) | -0.66 | -0.32 | 0.99 |  |  |
|  |  |  |  |  |  |
| **Colony density *B. lapidarius*** |  |  |  | 8 | 0.65 |
| Arable land (area) | 0.45 | 0.76 | 0.88 |  |  |
| Forestry (area) | -0.84 | -1.17 | 0.62 |  |  |
| Mass-flowering crops (area) | -0.24 | -0.29 | 0.99 |  |  |
| Artificial surfaces (area) | 0.72 | 1.30 | 0.53 |  |  |
| Field boundaries (length) | 0.11 | 0.23 | 0.99 |  |  |

* results presented here are minus the outlier. Due to small number of colony estimates, number of landscape parameters was reduced to three.
